# High Frequency Burst Firing of Granule Cells Ensures Transmission at the Parallel Fiber to Purkinje Cell Synapse at the Cost of Temporal Coding

**DOI:** 10.3389/fncir.2013.00095

**Published:** 2013-05-21

**Authors:** Boeke J. van Beugen, Zhenyu Gao, Henk-Jan Boele, Freek Hoebeek, Chris I. De Zeeuw

**Affiliations:** ^1^Department of Neuroscience, Erasmus MC RotterdamNetherlands; ^2^Netherlands Institute for Neuroscience, Royal Dutch Academy of Arts and SciencesAmsterdam, Netherlands

**Keywords:** cerebellum, synaptic transmission, parallel fiber, Purkinje cell, bursting activity, granule cell

## Abstract

Cerebellar granule cells (GrCs) convey information from mossy fibers (MFs) to Purkinje cells (PCs) via their parallel fibers (PFs). MF to GrC signaling allows transmission of frequencies up to 1 kHz and GrCs themselves can also fire bursts of action potentials with instantaneous frequencies up to 1 kHz. So far, in the scientific literature no evidence has been shown that these high-frequency bursts also exist in awake, behaving animals. More so, it remains to be shown whether such high-frequency bursts can transmit temporally coded information from MFs to PCs and/or whether these patterns of activity contribute to the spatiotemporal filtering properties of the GrC layer. Here, we show that, upon sensory stimulation in both un-anesthetized rabbits and mice, GrCs can show bursts that consist of tens of spikes at instantaneous frequencies over 800 Hz. *In vitro* recordings from individual GrC-PC pairs following high-frequency stimulation revealed an overall low initial release probability of ~0.17. Nevertheless, high-frequency burst activity induced a short-lived facilitation to ensure signaling within the first few spikes, which was rapidly followed by a reduction in transmitter release. The facilitation rate among individual GrC-PC pairs was heterogeneously distributed and could be classified as either “reluctant” or “responsive” according to their release characteristics. Despite the variety of efficacy at individual connections, grouped activity in GrCs resulted in a linear relationship between PC response and PF burst duration at frequencies up to 300 Hz allowing rate coding to persist at the network level. Together, these findings support the hypothesis that the cerebellar granular layer acts as a spatiotemporal filter between MF input and PC output (D’Angelo and De Zeeuw, 2009).

## INTRODUCTION

Understanding synaptic efficacy is a critical step toward unraveling the computational properties of a neuronal network. In the cerebellum, the cortical network is fed by two distinct inputs including mossy fibers (MFs) and climbing fibers (CFs), both known to fire action potentials at high frequencies paired with a high probability of vesicular release (Saviane and Silver, 2006; De Zeeuw et al., 2011). Even though both projections share these characteristics, each has a very distinct way of transmitting information to their postsynaptic targets; whereas CF-terminals display rapid vesicular depletion and lose synaptic power with consecutive action potentials (Schmolesky et al., 2005), MF-terminals are remarkably well equipped to facilitate reliable signaling at a high-frequency up to 1 kHz (Sargent et al., 2005; Saviane and Silver, 2006; Hallermann et al., 2010). Thus, while CFs and MFs both display high-frequency activity, in terms of functional implications they represent both ends of the high-frequency bursting spectrum, namely highly reliable non-graded signaling versus frequency dependent signaling, respectively.

Mossy fibers convey their information to the Purkinje cells via granule cells (GrCs), each of which provides a single ascending axon that bifurcates into a parallel fiber (PF). Like MF to GrC signaling, GrCs themselves can also fire bursts of action potentials (Eccles et al., 1966; Chadderton et al., 2004; Hensbroek et al., 2005; Jörntell and Ekerot, 2006). At rest they are rather silent, but following sensory activation GrCs display bursts of tens of action potentials with instantaneous frequencies up to 1 kHz (Isope and Barbour, 2002; Chadderton et al., 2004). As such GrCs may serve as a high-pass spatiotemporal filter, in which frequency dependent activity from MFs creates a time-window in which information can be relayed from MFs to PCs (D’Angelo and De Zeeuw, 2009; Mapelli et al., 2010; Solinas et al., 2010). This filter function probably results partly from Golgi cell inhibition (Brickley et al., 1996), but in principle additional filtering might occur at the PF-PC synapse. Indeed, the burst-like activity of GrCs may have two potential implications: (1) when paired with a high synaptic efficacy at the PF to PC input, it would allow GrCs to act as a relay, preserving frequency-coded information from MFs; and/or (2) when paired with a low synaptic efficacy, it could allow GrCs to filter individual inputs and signal only when strongly activated, albeit at the expense of temporal precision. When addressing the synaptic efficacy at the PF to PC input, the release probability (RP) is one of the main factors that should be taken into account. Yet, most variables that make up the RP, such as presynaptic calcium influx and the number of vesicles readily available for release, are influenced by preceding activity, and as a consequence, the RP becomes a dynamic variable within a high-frequency burst. Thus, the functional implications of PF bursts cannot be deduced from the initial RP alone and ongoing processes such as facilitation, depression and depletion should also be considered.

The burst-like activity in GrCs has been demonstrated in various anesthetized or decerebrated mammals (Eccles et al., 1966; Chadderton et al., 2004; Jörntell and Ekerot, 2006). However, it remains to be shown whether high-frequency GrC bursts can also be demonstrated in awake behaving animals (but see Hensbroek et al., 2005, 2006), and if so, to what extent high-frequency information can be conveyed onto PCs. This latter question is of importance because different results have been found for different strains of rats in this respect. For example, estimates of RP at the PF-PC synapse gathered in different rat strains using different experimental protocols range from 0.05 to 0.9 (Dittman et al., 2000; Isope and Barbour, 2002; Valera et al., 2012). Thus, in order to elucidate the potential role of high-frequency bursts in GrCs in spatiotemporal filtering of the cerebellar cortical network, we set out a series of experiments. We investigated the occurrence of bursting activity of GrCs by performing extracellular recordings in awake, behaving animals; we studied the impact of bursting activity in groups of PFs on excitatory postsynaptic currents (EPSCs) in PCs using whole cell recordings *in vitro*; and we examined the impact of a burst within a single PF on a PC using paired GrC – PC recordings.

## MATERIALS AND METHODS

### *IN VIVO* GrC RECORDING IN RABBIT

To give an example of GrC bursting activity in larger mammals as highlighted in the introduction, we present a recording of high-frequency activity in Dutch-belted rabbits, which have been investigated as partially discussed by Hensbroek et al. (2006). In short, extracellular recordings of cerebellar GrCs located in the flocculus were acquired from awake, behaving rabbits (3–6 months of age) using fine-tipped glass micro-electrodes (~1 μm tip diameter). Given the predominant silent behavior of GrCs at rest, cells were located while the animal was stimulated by rotation around the vertical axis. While characteristic spiking behavior was often suggestive of the cells subtype, further identification was confirmed by comparison of spontaneous spiking behavior to the algorithm as described (Ruigrok et al., 2011). Recordings were filtered below 100 Hz and above 3 kHz and sampled at 20 kHz (CED power 1401, Cambridge Electronic Design, UK).

### *IN VIVO* GrC RECORDINGS IN MICE

Extracellular recordings of cerebellar GrCs located in crus I were acquired from awake behaving C57Bl/6 wild-type mice (12–20 weeks of age) using glass microelectrodes (~0.5 μm tip diameter). Head-fixed mice were placed on top of a cylindrical treadmill that enabled the animal to walk freely during the experiment. Responsive GrCs were located while the animal was stimulated by a mild air puff to the whiskers (duration 10 ± 2 ms) every 10 s ipsilateral to the recording side. Identification was confirmed by comparison of spontaneous spiking behavior to the algorithm as described (Ruigrok et al., 2011). Recordings were filtered below 300 Hz and above 3 kHz and sampled at 25 kHz (RZ5 Tucker-Davis Technologies, Alachua, FL, USA).

### *IN VITRO* PATCH CLAMP RECORDINGS FROM PCs FOLLOWING GROUPED PF STIMULATION IN MICE

Sagittal slices (200–250 μm thickness) of the cerebellar vermis of adult male C57Bl/6 wild-type mice (8–30 weeks) were prepared in ice-cold artificial cerebrospinal fluid (aCSF) and stored at room temperature in carbogen-bubbled (95% O_2_ and 5% CO_2_) aCSF containing (in mM): 124 NaCl, 5 KCl, 1.25 Na_2_HPO_4_, 2 MgSO_4_, 2 CaCl_2_, 26 NaHCO_3_, and 10 D-glucose. Whole-cell patch clamp recordings were acquired 1–6 h after slice preparation from PCs using a HEKA EPC-10 amplifier (HEKA Electronics, Germany) at near physiological temperature (34 ± 1°C). Recording electrodes (2.5–4.0 MΩ) were filled with a solution containing (in mM): 9 KCl, 10 KOH, 3.48 MgCl_2_, 4 NaCl, 120 K-gluconate, 10 HEPES, 4 Na_2_ATP, 0.4 Na_3_GTP, and 28.5 sucrose (pH-adjusted to 7.25 ± 0.05). For experiments conducted in current clamp, the membrane-impermeable voltage gated sodium channel blocker QX314 was added to prevent generation of action potentials. Throughout recordings, slices were perfused with carbogen-bubbled aCSF to which picrotoxin (100 μM) was supplemented in order to isolate excitatory inputs. All drugs were acquired from Sigma-Aldrich, except γ-D-glutamylglycine (γDGG; Tocris). Recorded currents were filtered (low-pass Bessel, 3 kHz) and sampled at 20 kHz using Pulse-software (HEKA Electronics, Germany). PFs were activated by current injection (100 μs, 0.5–2.0 mA; ISO-flex current generator, A.M.P.I., Israel) using a bipolar glass microelectrode positioned in the molecular layer. Input and series resistance were monitored in each experiment and cells were rejected if a change of >10% occurred. In general, the stimulus protocol consisted of a sequence of high-frequency bursts of 2, 3, 4, 5, 10, 15, and 20 pulses. This sequence was tested with burst-frequencies varying between 100, 300, 500, and 700 Hz (with a 10, 3.33, 2, and 1.47 ms interstimulus interval, respectively) and repeated three times. Consecutive bursts were given at 0.05 Hz to minimize residual effects. For some experiments responses were also tested with bursts at 200 Hz (5 ms stimulus interval). To minimize the possibility of recruiting additional PFs with consecutive pulses in a burst and to promote reproducibility between recordings we applied several strategies: (1) a low-resistance bipolar stimulus electrode was used to reduce stimulus width and minimize current build-up within a burst keeping the stimulus region restricted; (2) the stimulus electrode was positioned close to the pial surface of the molecular layer where PF density is lowest; and (3) stimulus strength was adjusted to elicit a response of ~100 pA to a single stimulus. It is important to note that a limitation to signaling (see results) could only be demonstrated when careful attention was given to minimizing recruitment. However, residual recruitment cannot be ruled out completely and, in fact, is likely to have occurred to some degree.

### *IN VITRO* DOUBLE PATCH CLAMP RECORDINGS FROM GrC-PC PAIRS IN MICE

A double patch clamp configuration was favored over the “loose-cell-attached”-configuration (Isope and Barbour, 2002; Valera et al., 2012) to exclude the possibility of exciting more than a single GrC. Slices and electrodes were prepared under similar conditions as mentioned above. GrC patch pipettes had a resistance of 8–15 MΩ and contained: 126 K-Gluconate, 1 MgSO_4_, 4 NaCl, 5 HEPES, 0.05 CaCl_2_, 0.1 BAPTA, 15 D-Glucose, 3 MgATP, 0.1, and Na_3_GTP (pH 7.25–7.35). Experiments were performed at 34 ± 1°C. After the double patch clamp configuration was established, spike trains were elicited by somatic current injections for 50 ms in the GrC. The amplitude was adjusted such that spiking occurred at ~200 Hz. During the experiment connectivity was confirmed by eye when an EPSC was detected in the PC after averaging a minimum of 10 trains.

### ANALYSES

#### In Vivo recordings

Extracellular recordings from the rabbit were acquired using Spike 2 (Cambridge Electronic Design). Extracellular recordings from the mouse were acquired using open exe software by Tucker-Davis Technologies (Alachua, FL, USA). Data was analyzed using custom written routines in MATLAB (Mathworks). Spikes were separated upon waveform templates and values were exported to Excel (Microsoft) for further analysis.

#### PF group stimulation and in vitro recordings

Measurements of peak amplitude and charge were averaged over three recordings to minimize variance and then normalized to the response elicited by two pulses at 300 Hz. This particular response was chosen as a reference, because, generally, it would elicit the most consistent response and, therefore, allowed for better comparison between cells. For those experiments in which individual EPSC amplitudes were measured, the first derivative of the stimulus artifact was used to define intervals. Amplitudes were calculated as the difference between the local minimum and the current directly preceding the stimulus artifact. When stimuli took place within the rising phase of the preceding EPSC (which was especially prominent at the higher frequencies) and the response to the first pulse in the burst was more than 10% smaller than the response measured for a single stimulus, the recordings were excluded from analysis. During prolonged activation (burst length >100 ms) a plateau current could be observed. The level of this current was determined by averaging all values that preceded each individual stimulus artifact over a 50 ms time window.

#### Double patch granule cell – purkinje cell recordings

After connectivity was confirmed, high-frequency noise was eliminated offline from individual recordings using a running average (1 ms width). The recording was further processed by averaging the measured current over 800 μs creating virtual bins. Whenever the derivative of these values was negative over two or more consecutive bins, events were considered for analysis and both amplitude and derivative of the rising phase were measured. Baseline values of these parameters were determined over a 50 ms period preceding the current injection. Detected events were considered evoked responses when both amplitude and derivative exceeded 1 × SD over baseline levels and the detected amplitude rose 2 × SD above background noise levels. This method proved very effective to detect wider EPSCs while simultaneously rejecting high-frequency background noise. As an indicator of sensitivity, detected EPSC amplitudes were 5.2 ± 0.2 × SD larger than baseline events. Detection thresholds were -5.76 ± 0.32 pA and -5.9 ± 1.4 pA for ‘reluctant’ and ‘responsive’ pairs, respectively (see text); these values did not differ significantly (*p* > 0.05). For a given cell, failure rate (FR) was calculated for each individual stimulus number as the number of recordings in which no evoked response could be detected divided by the total number of recordings. RP was calculated as 1-FR. Note that RP indicates the chance that an action potential results in a detectable postsynaptic response (irrespective of its size) and does not reflect the probability of release for an individual vesicle (in the literature commonly described as “p”). Significance was tested using (un-)paired Student’s *t*-test or ANOVA where applicable. All values are expressed as average ± SEM unless otherwise noted.

## RESULTS

### BURSTING ACTIVITY *IN VIVO*

**FIGURE 1:**
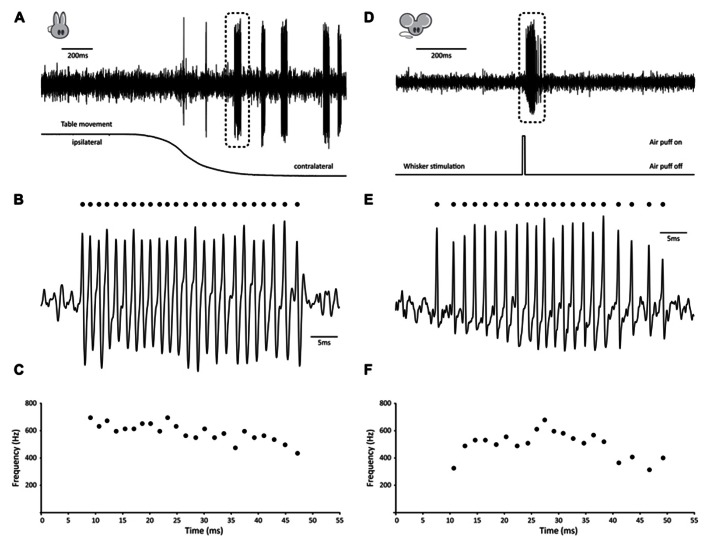
**Examples of burst firing in cerebellar granule cells**. **(A–C)** Example of high-frequency GrC burst in the awake behaving rabbit. **(A)** (top) Extracellular recording of granule cell in the flocculus of a Dutch-belted rabbit during vestibular stimulation around the vertical axis. Note absence of activity prior to movement onset, whereas several distinct, high-frequency bursts occur during rotation (table position is displayed at bottom). **(B)****Burst demarked in A shown at larger scale. Identified spikes are indicated by dots. **(C)** Instantaneous frequency plot of burst shown in B. This particular burst consisted of 24 consecutive spikes with instantaneous frequencies as high as 694 Hz and had an average firing frequency of 589 Hz. **(D–F)** Example of high-frequency GrC burst in the awake behaving mouse. **(D)** (top) Extracellular recording of granule cell in crus I of an awake mouse following whisker stimulation. Note absence of activity prior to movement onset, whereas a distinct, high-frequency burst occurs during stimulation. **(E)****Burst demarked in D shown at larger scale. Identified spikes are indicated by dots. **(F)** Instantaneous frequency plot of burst shown in E. This particular burst consisted of 21 consecutive spikes with instantaneous frequencies as high as 678 Hz and had an average firing frequency of 501 Hz.

Several observations have shown that GrCs can fire bursts of action potentials at surprisingly high frequencies of several 100 Hz with instantaneous frequencies up to 1 kHz (Chadderton et al., 2004; Hensbroek et al., 2005; Jörntell and Ekerot, 2006; for earlier observations in anesthetized cats see Eccles et al., 1966). Hensbroek et al. (2005) have been able to demonstrate that in the awake, behaving rabbit, GrCs remain predominantly silent at rest, but can generate bursts of action potentials of tens of spikes with average frequencies as high as 700 Hz during vestibular stimulation. Because, to the best of our knowledge, no recording of GrC activity in a behaving, un-anesthetized animal has been published in the scientific literature so far, we have included an example trace taken from the rabbit to illustrate these physiologically relevant bursting patterns (**Figures 1A–C**). This particular GrC did not show any activity when the animal was at rest, whereas vestibular stimulation via sigmoidal rotation around the vertical axis caused it to fire bursts of action potentials both during movement in the contralateral direction and while the animal was stationary in the contralateral position. A total of 31 bursts were fired over six cycles. Bursts had an average firing frequency of 529.8 ± 45.7 Hz, contained 11.6 ± 6.6 spikes and were 23.66 ± 15.86 ms in length (all values AVG ± SD). Surprisingly, for this cell, timing of burst onset was variable [1.85 ± 1.08 ms from start of movement (AVG ± SD), *n* = 6] and multiple bursts could occur within a single movement (5.17 ± 3.49 bursts per cycle; AVG ± SD). Due to this inconsistent behavior, bursting did not directly relate to either velocity, acceleration, position of the table or position of the eye, but rather seemed to signal movement in the contralateral direction indistinctively.

To confirm that similar activity patterns exist in awake, behaving mice, we performed extracellular recordings in crus I, while sensory stimulation was provided by applying a brief air puff to the whiskers (**Figures 1D–F**). In homology with the rabbit, the GrC shown here displayed very little activity at rest, whereas mild whisker stimulation with an air puff caused it to fire bursts of action potentials (**Figures 1D,E**). A single air puff to the whiskers could elicit multiple bursts, resulting in a total of 26 bursts identified over 15 trials. Bursts had an average firing frequency of 358.3 ± 82.9 Hz, but could reach instantaneous frequencies up to 815.9 Hz. They contained 10.8 ± 13.5 spikes and were 24.1 ± 26.5 ms in length (all values AVG ± SD). In addition, timing of burst onset was variable (28.08 ± 24.46 ms) from the start of whisker stimulation.

### IMPACT OF BURSTING ACTIVITY IN GROUPS OF PFs *IN VITRO* IN MICE

**FIGURE 2:**
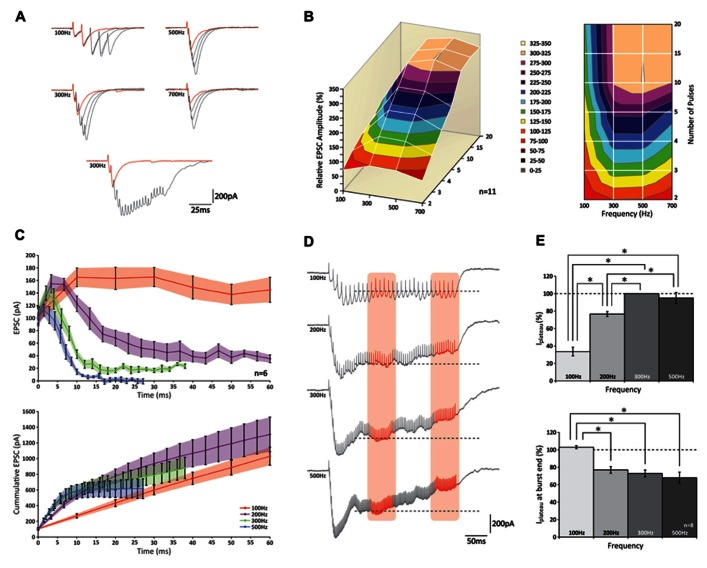
**PCs can increase the peak amplitude of their EPSCs following high-frequency stimulation of groups of PFs up to 300 Hz**. **(A)** (top) Traces of PC EPSCs following stimulus bursts of 2, 3, 4, or 5 stimuli at 100, 300, 500, or 700 Hz. Response to two stimuli is shown in red for reference. Note the effect of temporal summation on peak amplitude at higher frequencies. (bottom) Recordings of 2 and 20 stimuli at 300 Hz. Note how in the last half of the long burst no robust responses can be distinguished. **(B)** 3D representation (left) and top-down view (right) relative peak amplitudes of EPSCs with respect to different stimulus conditions. No increase in EPSC amplitude was observed for bursts of more than 10 stimuli at any of the stimulus frequencies (*p* > 0.05). **(C)** Relative EPSC increases per stimulus (top) and cumulative EPSC (bottom) over time. **(D)** Example traces of EPSCs following prolonged stimulation for 300 ms at 100, 200, 300, and 500 Hz. A plateau was established after ~100 ms for all frequencies. Average current levels were measured early (left orange column) and late (right orange column) during the plateau over a 50 ms time period. Levels measured during first period are indicated by dashed lines for reference. **(E)** (top) Bar graph of early plateau current levels; no significant increase was observed between 300 and 500 Hz (*p* < 0.05; *n* = 8). (bottom) Relative attenuation of plateau current levels at end of burst; no reduction was observed only at 100 Hz (*p* > 0.05).

Having confirmed that also in the mouse physiologically relevant activity patterns in GrCs consist of high-frequency bursts, we performed experiments to characterize postsynaptic responses evoked by stimulation of a bundle of PFs. We performed whole cell somatic patch-clamp recordings from PCs to measure EPSCs evoked by extracellular stimulation of groups of PFs. Bursts of 2, 3, 4, 5, 10, 15, and 20 pulses were given at frequencies of 100, 200, 300, 500, and 700 Hz (**Figure 2**; data obtained at 200 and 700 Hz are only shown in part of the panels). The peak amplitude of the EPSCs showed a significant increase with each additional stimulus (*p* < 0.05 for all frequencies; *n* = 11) until a maximum level was reached (**Figure 2A**); from 10 pulses on additional pulses did not contribute to a significant increase in peak amplitudes (*p* > 0.05 for all frequencies; *n* = 11; **Figure 2B**). Comparing bursts of equal numbers of stimuli over different frequencies, a significant increase in the relative peak amplitude of the EPSCs was seen (i.e., normalized to the response elicited by two pulses at 300 Hz) for each condition between 100 and 300 Hz (*p* < 0.05 for all numbers of stimulus pulses; *n* = 11; **Figure 2B**). No further effect on EPSC amplitudes could be observed for frequencies higher than 300 Hz (*p* > 0.05 for all numbers of stimulus pulses; *n* = 11) and in fact, the EPSCs following bursts of 2, 3, and 4 stimuli at 700 Hz were significantly smaller than those at 300 and 500 Hz (*p* < 0.05 for all comparisons; *n* = 11) possibly reflecting insufficient presynaptic calcium entry, which may occur at higher frequencies (Brenowitz and Regehr, 2007).

Temporal summation of consecutive EPSCs grossly affected the compound EPSC amplitude, in particular at higher frequencies. For example, at 100 Hz the second stimulus always occurred late in the decay phase of the preceding response, whereas at 500 Hz the second stimulus usually occurred around its peak (**Figure 2A**). To eliminate the effect of temporal summation and better reveal underlying processes of short-term plasticity, we looked at the response elicited by each individual stimulus within a burst. However, because stimuli could take place within the rise phase of the preceding EPSC (and thus underestimate the amplitude of the first response) many recordings had to be rejected from analysis (see methods). Of all the recordings that were included in the analyses none of the first EPSCs showed a significant attenuation (*p* > 0.05 for all frequencies; *n* = 6; **Figures 2A–C**).

Remarkably, while facilitation was observed during onset of the burst at all frequencies, such augmented responses could only be maintained at 100 Hz (**Figure 2C**, top graph); at higher frequencies a rapid decline was observed. Later in the burst (up to 20 stimuli), currents eventually reached a stable amplitude, but these levels reduced progressively at higher frequencies and at 700 Hz virtually no current could be detected. These levels all differed significantly from one another (*p* < 0.05 at 20 pulses for all comparisons; *n* = 6). When expressed as cumulative responses over burst duration (**Figure 2C**, bottom graph), the slope fitted to the first 10 ms grew significantly up to 300 Hz (*p* < 0.05 between frequencies of 100, 200, and 300 Hz; *n* = 6), while no additional rise was seen above 300 Hz (*p* > 0.05; *n* = 6).

Also when PFs were stimulated for a prolonged period of 300 ms, compound responses showed a distinct rising and decaying phase until a plateau was reached after approximately 100 ms (**Figure 2D**). Because a plateau establishes when both driving and restricting forces are at equilibrium, its level indicates the relative contribution of each force (Saviane and Silver, 2006). The amplitude of the steady-state level, which was measured as the average current recorded prior to each stimulus over a 50 ms period (period indicated by left red column in **Figure 2D**), was lowest for 100 Hz and significantly increased for bursts at 200–300 Hz (*p* < 0.05; *n* = 8; **Figure 2E**, top graph). No further increase was observed between 300 and 500 Hz (*p* > 0.05; *n* = 8), indicating that the driving force showed no further increase above 300 Hz. As stimulation continued beyond 150 ms, the plateau could be maintained up to 300 ms for bursts at 100 Hz, whereas at the higher frequency levels it showed a significant reduction toward the end of the burst (*p* < 0.05; *n* = 8; **Figure 2E**). Remarkably, whereas at 200 Hz the steady-state had not reached its maximum yet, we saw a reduction of the plateau toward the end of the stimulus, pointing toward a presynaptic origin of this insufficiency. While these results show that release is optimized around 300 Hz, it is important to note that frequency-dependent limitation of release and presynaptic insufficiency probably occur at frequencies lower than reported here, because the results above apply to PF-activity *as a bundle*; in this experimental configuration inactivity of a particular fiber can be compensated for by activity from others, and unless all fibers are continuously active, equilibrium can be established at a higher level than would be possible for fibers independently. Indeed, this tenet is supported by our double-patch recordings (see below) indicating that individual PFs cannot maintain sustained release.

**FIGURE 3:**
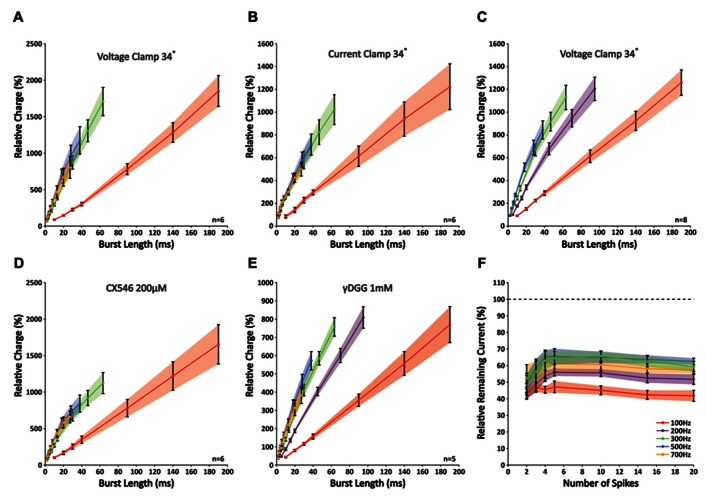
**Slopes of nearly perfect linear correlations between charge and burst length reveal limited signaling for frequencies higher than 300 Hz as a result of insufficient glutamate release**. **(A)** Correlations between relative charge and burst length at 100, 300, 500, and 700 Hz; no differences were seen in the slopes between 300, 500, and 700 Hz (*p* > 0.05; *n* = 6). **(B)****Similar experiment as shown in A repeated in current clamp. Recording conditions had no effect on the outcome (*n* = 6). **(C)** Similar experiment as shown in A, now including 200 Hz. Note that the slope measured for 200 Hz differed significantly from that at 100, 300, and 500 Hz (*p* < 0.05; *n* = 8). **(D)** When CX546 was applied, correlations were less linear as a result of small facilitations for bursts smaller than 20 ms (see text). However, bursts that were shorter than 20 ms in duration still displayed a linear relationship and the slopes at 300, 500, and 700 Hz did not differ significantly from each other (*p* > 0.05; *n* = 6). **(E)** Application of γDGG (1 mM) did not affect the observed limitation of signaling at frequencies higher than 300 Hz (*n* = 5). **(F)** γDGG (1 mM) effectively suppressed all responses by more than 35%. Relative suppression was significantly smaller after five stimuli at 200, 300, and 500 Hz, although no further decrease was observed for longer stimulus trains. This indicates that release might be facilitated within the first few stimuli, but quickly reaches its optimum (*n* = 5).

While the analysis of peak amplitudes can describe the physiologically relevant effect of temporal summation within a compound response to high-frequency activity, this approach cannot fully describe the properties of synaptic release, because other processes, such as postsynaptic receptor saturation and voltage-sensitive repolarizing currents, can potentially mask any ongoing release. Therefore, to get a better understanding of the total amount of neurotransmitter released throughout a burst, we looked at the total charge (i.e., “area-under-the-curve” of an EPSC). Correlating the total charge to burst duration revealed a remarkable nearly perfect linear fit for all frequencies (*R*^2^-values 0.990 ± 0.002; *n* = 6; **Figure 3A**). The slope increased significantly with a rise in frequency for 100 and 300 Hz (*p* < 0.05 for all combinations; *n* = 6), which indicates that the stimulated PF bundle sustains the release at these frequencies. However, in accordance with our observations described above, no further rise in the slope was found for frequencies above 300 Hz (*p* < 0.05 for all frequencies; *n* = 6). This finding indicates that, within a given time period, stimuli at frequencies higher than 300 Hz are ineffective in eliciting any additional release. These “false” stimuli occur at a rate faster than a bundle of PFs can compensate for. Considering the compensatory mechanism of alternate activity that occurs within a group of fibers, the maximum frequency at which individual fibers can signal is likely lower. These results are a first indication that GrC burst firing at frequencies higher than 300 Hz cannot relay temporally coded information from MFs to PCs. To exclude the possibility that these findings resulted from our whole-cell voltage-clamp recording conditions, we repeated the experiment in current-clamp mode so as not to restrict the flow of electrical currents in PCs. Again, no differences were found at higher frequencies (*p* < 0.05 for 100 Hz against 300, 500, and 700 Hz, *p* > 0.05 between 300, 500, and 700 Hz; *n* = 6; **Figure 3B**). To confirm that a transitional trajectory existed before the observed limit of 300 Hz was reached, we also included bursts of 200 Hz (**Figure 3C**). The slope measured for 200 Hz was indeed larger than 100 Hz (*p* < 0.05; *n* = 8), yet smaller than 300–500 Hz (*p* < 0.05; *n* = 8).

To detect any involvement of postsynaptic receptor desensitization on signaling, we applied CX546, which acts as an allosteric modulator that prevents α-amino-3-hydroxy-5-methyl-4-isoxazolepropionic acid receptors (AMPARs) to reside in the desensitized state (Lynch and Gall, 2006). With CX546 present (200 μM) the data did not fall along a linear fit as perfectly as without (*R*^2^-values 0.974 ± 0.004; *n* = 6), due to a small facilitation in responses to bursts shorter than 20 ms in duration (**Figure 3D**). While hypothetically this finding could also be explained by depression of longer bursts, our results from both the γDGG experiments and the double-patch GrC-PC recordings (see below) confirm that initial facilitation followed by rapid depletion underlie these findings. Still, over the first 20 ms the slope with CX546 displayed a linear relation (*R*^2^-values 0.989 ± 0.002; *n* = 6) and differed only significantly at 100 Hz (*p* < 0.05; *n* = 6). No differences were found for the remaining frequencies (*p* > 0.05; *n* = 6). While these results suggest some role of AMPAR desensitization in PF-PC signaling, it cannot explain the restrictions in signaling found for stimulus frequencies over 300 Hz.

Next, to exclude any major involvement of postsynaptic receptor saturation we investigated the impact of γDGG, which is a competitive antagonist of AMPARs that effectively suppresses EPSCs by occupying a portion of the available AMPARs. Because of its competitive behavior and fast unbinding rate, the relative suppression by γDGG is a perfect reporter of glutamate transients; in the case of postsynaptic receptor saturation, excessive glutamate will compete with γDGG to overcome its suppressive effect and EPSCs will be unaffected by its presence. At 1 mM, γDGG effectively suppressed all responses by more than 35% of their original size. However, no effect was observed on the linear behavior of responses (*R*^2^-values 0.993 ± 0.001; *n* = 5). In addition, in accordance with our earlier results, slopes were significantly lower at 100 and 200 Hz compared with other frequencies (*p* < 0.05; *n* = 5), yet those at 300, 500, and 700 Hz were indistinguishable from each other (*p* > 0.05; *n* = 5; **Figure 3E**). Interestingly, the relative reduction was strongest for short bursts, but showed a gradual decrease toward bursts of five pulses at 200, 300, and 500 Hz (*p* < 0.05 for responses of two versus five pulses at all three frequencies; *n* = 5; **Figure 3F**), indicative of additional glutamate release with consecutive pulses. However, no further decrease was observed for longer bursts (*p* < 0.05 for responses of five versus 20 pulses; *n* = 5), meaning that release had reached its optimum within five pulses. Together, these results indicate that signaling at high-frequency between PF-PC is restricted to the presynaptic site by insufficient release. While some additional release might occur within the first few pulses of a burst, prolonged activation cannot be maintained. Also, despite the facilitation we found for individual EPSCs at 100 Hz (**Figure 2C**), this was not accompanied by a shift in the relative glutamate content per synapse (**Figure 3F**). Therefore, the reported facilitation mostly reflects recruitment of more synapses and not additional transmitter release at individual synapses, highlighting that results obtained through stimulation of a bundle of PFs cannot be extrapolated directly to the level of individual synapses. More so, our data show that overall synaptic release is not facilitated at 100 Hz.

### PAIRED RECORDINGS OF GrCs and PCs *IN VITRO*

The results described above showed the implications of grouped activity from synchronously activated GrCs, but they failed to accurately describe the behavior of individual connections. We therefore performed paired whole-cell patch clamp recordings from 13 connected GrC – PC pairs in mice. Because this configuration turned out to be relatively short-lived, we could not test responses for the same wide range of stimuli as with extracellular stimulation. We chose to elicit bursts of action potentials at 200 Hz, because at this frequency, on the one hand, we found some limitations such as depletion with prolonged activity, while on the other hand we observed potential room for additional signaling at higher frequencies (up to 300 Hz; see above).

**FIGURE 4:**
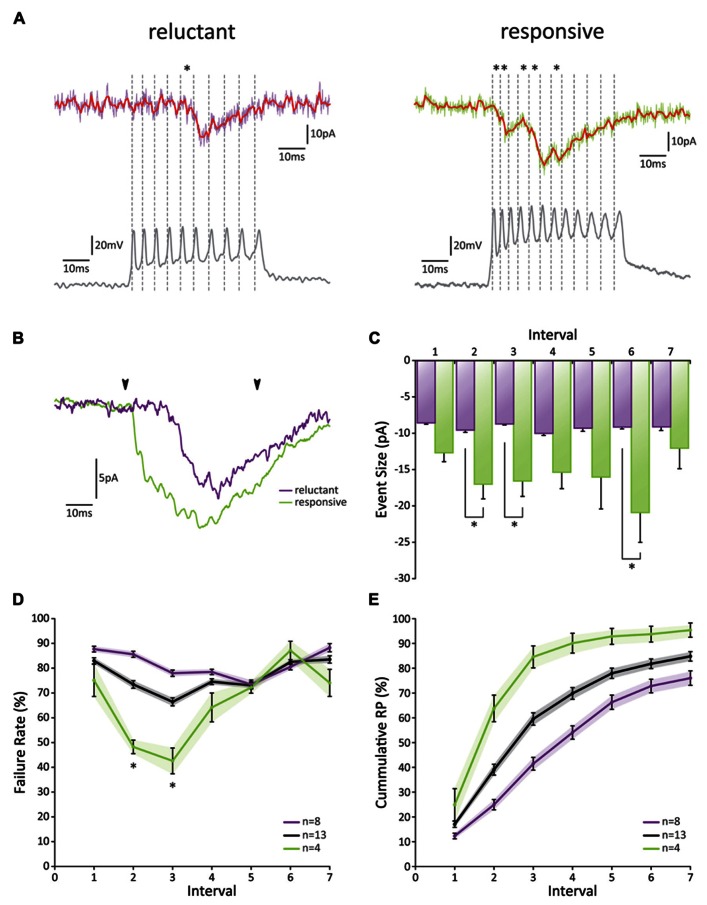
**Paired GrC-PC recordings reveal heterogeneity in PF release probability**. **(A)** Examples of paired recordings. Based on the cumulative release probability over the first three spikes, pairs could be subdivided into a “reluctant” group (left) and “responsive” group (right). (top traces) Original voltage clamp recordings from PCs (purple and green for “reluctant” and “responsive” connections, respectively) and their processed signals used for analysis (red); asterisks indicate detected events. (bottom traces) Current clamp recordings from GrCs. Spiking is induced via current injection. Dashed lines indicate detected spikes. **(B)** Averaged responses over all recordings from cells shown in A. Beginning and end of current injections are indicated by arrowheads. Note a faster overall response in the responsive connection (green). **(C)** EPSC amplitudes did not change in size during the burst for reluctant connections (purple, *p* > 0.05 for all comparisons). However, for responsive connections (green) EPSCs elicited by the 2nd, 3rd, and 6th spike were significantly larger than that by the 1st spike. No significant difference was observed between reluctant and responsive connections for EPSCs following the first spike. **(D)****Failure rate for all recorded pairs (gray), reluctant (purple), and responsive (green) connections. Responsive connections showed a short-lived facilitation for the 2nd and 3rd spike, resulting in a significantly smaller failure rate compared to reluctant connections (*p* < 0.05; *n* = 8 and *n* = 4, respectively; one cell out of the 13 was ambiguous and not included in one of the two main categories). **(E)** Cumulative probability of release for all recorded pairs (gray), reluctant (purple), and responsive (green) connections.

Recordings from individual GrC-PC pairs confirmed a low initial RP, yet substantial differences were observed between these pairs (**Figures 4A–D**). Overall, we found an initial failure rate (FR) of 0.83 ± 0.01 (**Figure 4D**). The FR was significantly reduced to a minimum of 0.66 ± 0.02 at the third action potential (*p* < 0.05; *n* = 13). However, from the third action potential on, the FR began to show a gradual increase again and returned to baseline levels at the sixth action potential (*p* > 0.05 1st versus 6th action potential, *p* < 0.05 3rd versus 6th action potential; *n* = 13). On the basis of the cumulative RP calculated over the first 3 action potentials [RP_cumulative 1-3_ = 1-(FR_1_^⋆^FR_2_^⋆^FR_3_)], pairs could be separated into a “reluctant”-group (RP_cumulative 1-3_ < 0.55, average RP_cumulative 1-3_ = 0.41 ± 0.05; *n* = 8) and a “responsive”-group (RP_cumulative 1-3_ > 0.8, average RP_cumulative 1-3_ = 0.86 ± 0.02; *n* = 4; **Figure 4A**). Even though the initial FR was similar between the two groups (*p* > 0.05; *n* = 8 and *n* = 4), a dramatic difference was observed between the FR of the 2nd and 3rd spike (*p* > 0.05, 0.86 ± 0.01 and 0.78 ± 0.01 for reluctant connections; 0.48 ± 0.03 and 0.43 ± 0.05 for responsive connections, respectively; *n* = 8 and *n* = 4, respectively; **Figure 4D**). No difference in FR was observed after the 3rd spike (*p* > 0.05; *n* = 8 and *n* = 4). Accordingly, the cumulative RP (RP_cumulative 1-n_ = 1-(FR_1_^⋆^FR_2_^⋆^FR_n_), unveiled a striking difference between the two groups (**Figure 4E**); whereas the chance of release for at least one of the action potentials reached >90% for the responsive connections at the fourth action potential this chance grew to ~50% for reluctant connections and had only reached ~75% at the 7th action potential.

The EPSC amplitude of the response to the first action potential was 8.54 pA ± 0.22 (*n* = 8) for reluctant connections (**Figure 4C**). No further change in amplitude occurred throughout the burst. Amplitudes for responsive connections were significantly larger for the 2nd, 3rd, and 6th action potential (*p* < 0.05; *n* = 4) and also responses to the 4th and 5th spike followed this trend. Thus, although the initial RP is low at PF-PC synapses, we conclude that high-frequency burst activity may facilitate release within the first few spikes (both in reluctant and responsive connections) and result in a high cumulative RP (in particular in responsive connections) to ensure signaling and overcome a low initial RP.

## DISCUSSION

For a long time, it was believed that cerebellar GrCs operate at low firing frequencies (but see Eccles et al., 1966). It is only due to recent technical advances that GrCs have been shown to fire bursts of action potentials at surprisingly high frequencies of several 100 Hz with instantaneous frequencies up to 1 kHz (Chadderton et al., 2004; Hensbroek et al., 2006; Jörntell and Ekerot, 2006; Valera et al., 2012) and that individual action potentials in high-frequency bursts of GrCs are reliably translated into consistent calcium transients at presynaptic PF varicosities, showing only minor attenuation for intervals starting at approximately 500 Hz (Brenowitz and Regehr, 2007). Here, we demonstrate that mice can also show bursts of GrC activity at high-frequency, but that the capability of their PFs to relay frequency coded information to PCs is limited to ~300 Hz for grouped activity. Moreover, at the level of individual PFs, release is too unreliable to convey temporally coded information. In line with previous studies in rat (Brenowitz and Regehr, 2007; for review see Le Guen and De Zeeuw, 2010), murine PF inputs constitute a heterogenic group of terminals with various levels of RP allowing differential filtering. Our data suggest that high-frequency PF activity may facilitate transmitter release during initial spiking to ensure signaling onto PCs, but that variability is too high to allow temporally coded signaling at individual synapses.

### LIMITATIONS OF TRANSMITTER RELEASE AT THE MURINE PF-PC SYNAPSE

Our main finding demonstrates that release from PF terminals onto PC dendrites is limited during physiologically relevant high-frequency bursts. Although the current observations in awake behaving mice and those of others in rabbits and anesthetized rats indicate that cerebellar GrCs can fire bursts of action potentials with instantaneous frequencies up to ~1 kHz (Chadderton et al., 2004; Hensbroek et al., 2005; Ruigrok et al., 2011), our *in vitro* results following stimulation of bundles of PFs show that signaling at the murine PF-PC synapse is confined to ~300 Hz at best. This maximal limit is likely to be an overestimation, because the stimulus conditions under which these results were obtained allow for different PFs to be active at different time points within a burst. In this manner, a group of PFs can maintain a highly effective activity pattern, while the effective activity of each individual terminal is in fact substantially lower (see also below). Indeed, our double patch-clamp recordings of connected GrC-PC pairs in mice confirmed that overall PF terminals exhibit a low RP. These recordings showed a low average initial RP of ~0.17, which is in line with previous observations obtained in rats by Dittman et al. (2000), but not by those of Valera et al. (2012), who reported an initial RP of 0.44. The differences with the latter study may be partially due to the specific strains of rodents used, differences in extracellular calcium concentration in the bath, and/or the approach used to calculate the RP. Valera et al. (2012) calculated RP with the multi-probability-fluctuation-analysis (MPFA), a method that relies on a presumed distribution of Pr_site_ (i.e., the probability for a single vesicle to be released at a particular site) to calculate RP from the quantal distribution. However, this approach does not take into account heterogeneity among synapses that arises from differential factors such as presynaptic calcium transients and/or build-up of residual calcium (Brenowitz and Regehr, 2007). Moreover, whereas Valera et al. (2012) used a noise-based signal-to-noise ratio to discriminate events in paired recordings, we applied a noise-based multi-variable threshold detection method; both methods have the potential to misrepresent RP either by missing small events or detecting noise, resulting in an underestimation or overestimation, respectively.

### PF TERMINALS IN MICE ARE HETEROGENIC

We noticed a clear heterogeneous distribution between release properties of individual connected GrC-PC pairs. Given that larger EPSC amplitudes coincided with a higher RP, this difference might reflect diversity between potentiated and depressed synapses (Bender et al., 2009). However, we cannot exclude the possibility that this heterogeneity also results partly from differences between terminals from the ascending versus the horizontal segment of PFs (Sims and Hartell, 2005, 2006). In addition, the heterogeneity may be related to the great diversity found in presynaptic calcium transients of PF terminals, which can differ more than 10-fold (Brenowitz and Regehr, 2007). It is not unlikely that this heterogeneity has resulted from experience-driven long-term plasticity, either facilitating or repressing the responsiveness of connections by adjusting presynaptic calcium transients.

Despite a heterogeneous distribution, we found a low initial RP for all PF-PC synapses; whereas the PF to Golgi cell synapse is considered to be weak (Dieudonné, 1998; Robberechts et al., 2010), the PF to molecular layer interneuron synapse has been shown to be more reliable (Crowley et al., 2007; Satake et al., 2012). These observations are remarkable, because all types of PF connections mentioned above probably persist along a single PF (Palay and Chan-Palay, 1974; Napper and Harvey, 1988), which inherently suggests differential filtering. Therefore, it will be intriguing to see how heterogeneity will affect the ability of PFs to signal at high frequencies, not only at their input to PCs, but also to Golgi cells and molecular layer interneurons. Moreover, it will be interesting to find out how the plasticity rules that control the efficacy at PF-PC synapses compare to those controlling PF-interneuron synapses (Gao et al., 2012).

### TECHNICAL LIMITATIONS AND CAVEATS

We took three different approaches in the current study to investigate GrC–PC interactions; these included extracellular recordings of GrCs *in vivo*, recordings of intracellular PC responses *in vitro* following extracellular stimulation of bundles of PFs, and intracellular recordings of individual GrC-PC pairs *in vitro*. The *in vitro* approaches presented particular technical limitations and caveats. Recording EPSCs from PCs following PF activity is made notoriously complicated by several factors (Roth and Häusser, 2001): the large dimension of a PC prevents the desired control over electrical properties in patch-clamp experiments; the extensive dendritic arborization filters postsynaptic currents; the small size and condense packing of PFs make isolated activation difficult; and spontaneously active inputs obscure individually evoked events. These complications probably had some effect on the measurements following PF bundle stimulations as well as those during the paired recordings. When stimulating a bundle of PFs, the elicited response will be the multiplication of dynamic stochastic variables and, given a relatively low RP, will only reflect activity from a subset of all the stimulated PFs. Considering the heterogenic behavior, when some fibers might show activity to a substantial portion of the stimuli in a burst, while others might respond only periodically, the composed response will not reflect activity from a constant number of terminals. Because the total number of stimulated fibers is unknown, it is unclear what proportion is unresponsive. When, for example, a burst of 10 stimuli is given, the response to the 4th and 5th stimulus can be made up by a completely different subset of PFs. As a result, heterogeneity greatly limits the possibilities for analysis of those responses elicited by grouped activity and, in fact, limits applicability of methods proven effective at other release sites (Saviane and Silver, 2007; Valera et al., 2012). Yet, an equilibrium between driving and suppressing forces will establish as heterogenic differences and asynchronous activation of fibers will be averaged out at least partly by sustained activity. Thus, the measurements following extracellular bundle stimulation may to some degree be subject to misrepresentation, but the steady-state current can probably serve as an indirect indicator of overall synaptic efficacy (Saviane and Silver, 2006; Valera et al., 2012). Finally, finding connected pairs of GrCs and PCs was indeed difficult and holding on to both cells for long periods of time proved even more challenging. These technical difficulties forced us to restrict ourselves to investigate only the most physiologically relevant stimulus parameters and limited the power of our statistical analyses.

### FUNCTIONAL IMPLICATIONS

The notion that frequency coding is partially lost in individual GrC firing patterns has interesting implications for the cerebellar network as a whole and challenges the idea that GrCs merely act as interposed relay-neurons. What is the purpose for a GrC to fire a high-frequency burst when actual release falls behind? The main benefit of a relatively low RP is that few spontaneous events occur, creating a relatively noise-free environment. However, this comes at the expense of reliability and consistency; such a system is not well-designed to employ rate coding over its entire frequency range in a linear fashion. Nevertheless, as depicted in **Figure 4**, the cumulative probability of release at the murine PF-PC synapse reached nearly 1 within a few spikes for the “High RP”-group. This means that a brief PF burst could overcome the initial low RP to ensure release within the time window of the burst. Moreover, as the presynaptic insufficiency caused a rapid fall in RP, restricted release prevents immediate saturation of the postsynaptic site, thus leaving room for temporal summation at a lower rate. Ultimately, these characteristics point toward a non-linear mode of synaptic transmission, in which the actual occurrence of a synaptic event bears significance as well as its timing within a burst.

Granule cell activity is tightly controlled by tonic inhibition from Golgi cells, resulting in few action potentials at rest (Hensbroek et al., 2006; Mapelli and D’Angelo, 2007); GrCs can be relieved from this inhibition when Golgi cell activity is diminished or when excitatory MF input exceeds the inhibiting force. Moreover, glutamate released by MFs can directly act on Golgi cell terminals and suppress GABA release, forming an activity-dependent feed-forward loop (Gao et al., 2012). When the balance is shifted from inhibition to excitation, a time window is created in which a GrC can fire a burst of action potentials (D’Angelo and De Zeeuw, 2009; Mapelli et al., 2010; Solinas et al., 2010). As such, the granular layer can be regarded as a “gate-keeper” that can selectively allow information to pass from MFs to PCs. Because MF terminals are tailored to maintain reliable signaling at very high frequencies (Sargent et al., 2005; Hallermann et al., 2010), it is remarkable that, to some extent, rate coding is lost further down the network. This implies that the information encoded by high-frequency firing of MFs may have limited value for PCs, but rather shapes the time window in which GrCs can produce a burst of activity. Thus, combining strong inhibition together with a relatively low RP results in a system in which synaptic events are restricted and a high signal-to-noise ratio is effectuated.

Our finding that bundles of PFs can display a nearly linear, frequency-sensitive relationship between burst duration and total synaptic charge has some physiological relevance, because there is evidence that PFs are activated in bundles (Ebner et al., 2005). Moreover, GrCs are also prone to fire together in groups, because of the impact of Golgi cell inhibition, which can produce a center-surround pattern of activity in the granular layer further enhancing the filter function of the granular layer (Mapelli and D’Angelo, 2007). This leads to the interesting possibility that, while output from individual PFs can be relatively insignificant and poorly timed, a group of selectively activated PFs can reliably convey and maintain frequency coded MF activity, while background noise from spontaneous release can be minimized.

In conclusion, our findings indicate that at the PF-PC synapse the firing mode of GrCs in high-frequency bursts overcomes the unreliability and inconsistency this synapse exhibits so as to ensure signaling at the partial cost of rate coding. Together with strong Golgi cell inhibition and center-surround activation of PF groups, this creates an environment in which the granular layer forms a strong spatiotemporal filter and, while a single GrC action potential becomes insignificant, controlled bursting can reliably convey selective information from MF input to PC output.

## Conflict of Interest Statement

The authors declare that the research was conducted in the absence of any commercial or financial relationships that could be construed as a potential conflict of interest.

## References

[B1] Bender V. A. Pugh J. R. Jahr C. E. (2009). Presynaptically expressed long-term potentiation increases multivesicular release at parallel fiber synapses. *J. Neurosci.* 29 10974–10978 19726655 10.1523/JNEUROSCI.2123-09.2009 PMC2775459

[B2] Brenowitz S. D. Regehr W. G. (2007). Reliability and heterogeneity of calcium signaling at single presynaptic boutons of cerebellar granule cells. *J. Neurosci.* 27 7888–7898 17652580 10.1523/JNEUROSCI.1064-07.2007 PMC6672738

[B3] Brickley S. G. Cull-Candy S. G. Farrant M. (1996). Development of a tonic form of synaptic inhibition in rat cerebellar granule cells resulting from persistent activation of GABAA receptors. *J. Physiol.* 497(Pt 3) 753–759 10.1113/jphysiol.1996.sp021806 PMC1160971 9003560

[B4] Chadderton P. Margrie T. W. Hausser M. (2004). Integration of quanta in cerebellar granule cells during sensory processing. *Nature* 428 856–860 15103377 10.1038/nature02442

[B5] Crowley J. J. Carter A. G. Regehr W. G. (2007). Fast vesicle replenishment and rapid recovery from desensitization at a single synaptic release site. *J. Neurosci.* 27 5448–5460 17507567 10.1523/JNEUROSCI.1186-07.2007 PMC6672343

[B6] D’Angelo E. De Zeeuw C. I. (2009). Timing and plasticity in the cerebellum: focus on the granular layer. *Trends Neurosci.* 32 30–40 18977038 10.1016/j.tins.2008.09.007

[B7] De Zeeuw C. I. Hoebeek F. E. Bosman L. W. Schonnewille M. Witter L. Koekkoek S. K. (2011). Spatiotemporal firing patterns in the cerebellum. *Nat. Rev. Neurosci*. 12 327–344 21544091 10.1038/nrn3011

[B8] Dieudonné S. (1998). Submillisecond kinetics and low efficacy of parallel fibre-Golgi cell synaptic currents in the rat cerebellum. *J. Physiol.* 510(Pt 3) 845–866 9660898 10.1111/j.1469-7793.1998.845bj.x PMC2231065

[B9] Dittman J. S. Kreitzer A. C. Regehr W. G. (2000). Interplay between facilitation, depression, and residual calcium at three presynaptic terminals. *J. Neurosci.* 20 1374–1385 10662828 10.1523/JNEUROSCI.20-04-01374.2000 PMC6772383

[B10] Ebner T. J. Chen G. Gao W. Reinert K. (2005). Optical imaging of cerebellar functional architectures: parallel fiber beams, parasagittal bands and spreading acidification. *Prog. Brain Res.* 148 125–138 15661186 10.1016/S0079-6123(04)48011-X

[B11] Eccles J. C. Llinás R. Sasaki K. (1966). The mossy fibre-granule cell relay of the cerebellum and its inhibitory control by Golgi cells. *Exp. Brain Res*. 1 82–101 5910945 10.1007/BF00235211

[B12] Gao Z. van Beugen B. J De Zeeuw C. I. (2012). Distributed synergistic plasticity and cerebellar learning. *Nat. Rev. Neurosci*. 13 619–635 22895474 10.1038/nrn3312

[B13] Hallermann S. Fejtova A. Schmidt H. Weyhersmüller A. Silver R. A. Gundelfinger E. D. (2010). Bassoon speeds vesicle reloading at a central excitatory synapse. *Neuron* 68 710–723 21092860 10.1016/j.neuron.2010.10.026 PMC3004039

[B14] Hensbroek R. A. Ruigrok T. J. H. van Beugen B. J. Simpson J. I. (2005). Burst modulation of cerebellar granule cells during vestibular stimulation. *Soc. Neurosci. Abstr.* 297 4

[B15] Hensbroek R. A. van Beugen B. J. Ruigrok T. J. H. Simpson J. I. (2006). Spike modulation of unipolar brush cells and granule cells in the awake rabbit. *Soc. Neurosci. Abstr.* 740 2

[B16] Isope P. Barbour B. (2002). Properties of unitary granule cell–>Purkinje cell synapses in adult rat cerebellar slices. *J. Neurosci.* 22 9668–9678 12427822 10.1523/JNEUROSCI.22-22-09668.2002 PMC6757845

[B17] Jörntell H. Ekerot C. F. (2006). Properties of somatosensory synaptic integration in cerebellar granule cells in vivo. *J. Neurosci.* 26 11786–11797 17093099 10.1523/JNEUROSCI.2939-06.2006 PMC6674774

[B18] Le Guen M. C De Zeeuw C. I. (2010). Presynaptic plasticity at cerebellar parallel fiber terminals. *Funct. Neurol.* 25 141–151 21232210

[B19] Lynch G. Gall C. M. (2006). Ampakines and the threefold path to cognitive enhancement. *Trends Neurosci.* 29 554–562 16890999 10.1016/j.tins.2006.07.007

[B20] Mapelli J. D’Angelo E. (2007). The spatial organization of long-term synaptic plasticity at the input stage of cerebellum. *J. Neurosci*. 27 1285–1296 17287503 10.1523/JNEUROSCI.4873-06.2007 PMC6673576

[B21] Mapelli J. Gandolfi D D’Angelo E. (2010). High-pass filtering and dynamic gain regulation enhance vertical bursts transmission along the mossy fiber pathway of cerebellum. *Front. Cell. Neurosci* 4: 14 10.3389/fncel.2010.00014 PMC2889686 20577586

[B22] Napper R. M. Harvey R. J. (1988). Number of parallel fiber synapses on an individual Purkinje cell in the cerebellum of the rat. *J. Comp. Neurol.* 274 168–177 3209740 10.1002/cne.902740204

[B23] Palay S. L. Chan-Palay V. (1974). *Cerebellar Cortex: Cytology and Organization*. Berlin: Springer

[B24] Robberechts Q. Wijnants M. Guigliano M De Schutter E. (2010). Long-term depression at parallel fiber to Golgi cell synapses. *J. Neurophysiol*. 104 3413–3423 20861429 10.1152/jn.00030.2010 PMC3007626

[B25] Roth A. Häusser M. (2001). Compartmental models of rat cerebellar Purkinje cells based on simultaneous somatic and dendritic patch-clamp recordings. *J. Physiol.* 535(Pt 2) 445–472 10.1111/j.1469-7793.2001.00445.x PMC2278793 11533136

[B26] Ruigrok T. J. Hensbroek R. A. Simpson J. I. (2011). Spontaneous activity signatures of morphologically identified interneurons in the vestibulocerebellum. *J. Neurosci.* 31 712–724 21228180 10.1523/JNEUROSCI.1959-10.2011 PMC6623423

[B27] Sargent P. B. Saviane C. Nielsen T. A. DiGregorio D. A. Silver R. A. (2005). Rapid vesicular release, quantal variability, and spillover contribute to the precision and reliability of transmission at a glomerular synapse. *J. Neurosci.* 25 8173–8187 16148225 10.1523/JNEUROSCI.2051-05.2005 PMC6725539

[B28] Satake S. Inoue T. Imoto K. (2012). Paired-pulse facilitation of multivesicular release and intersynaptic spillover of glutamate at rat cerebellar granule cell-interneurone synapses. *J. Physiol.* 590(Pt 22) 5653–5675 22930264 10.1113/jphysiol.2012.234070 PMC3528983

[B29] Saviane C. Silver R. A. (2006). Fast vesicle reloading and a large pool sustain high bandwidth transmission at a central synapse. *Nature* 439 983–987 16496000 10.1038/nature04509

[B30] Saviane C. Silver R. A. (2007). Estimation of quantal parameters with multiple-probability fluctuation analysis. *Methods Mol. Biol.* 403 303–317 18828002 10.1007/978-1-59745-529-9_19 PMC6129173

[B31] Schmolesky M. T. De Zeeuw C. I. Hansel C. (2005). Climbing fiber synaptic plasticity and modifications in Purkinje cell excitability. *Prog. Brain Res.* 148 81–94 15661183 10.1016/S0079-6123(04)48008-X

[B32] Sims R. E. Hartell N. A. (2005). Differences in transmission properties and susceptibility to long-term depression reveal functional specialization of ascending axon and parallel fiber synapses to Purkinje cells. *J. Neurosci.* 25 3246–3257 15788782 10.1523/JNEUROSCI.0073-05.2005 PMC6725092

[B33] Sims R. E. Hartell N. A. (2006). Differential susceptibility to synaptic plasticity reveals a functional specialization of ascending axon and parallel fiber synapses to cerebellar Purkinje cells. *J. Neurosci.* 26 5153–5159 16687506 10.1523/JNEUROSCI.4121-05.2006 PMC6674255

[B34] Solinas S. Nieus T D’Angelo E. (2010). A realistic large-scale model of the cerebellum granular layer predicts cicuit spatio-temporal filtering properties. *Front. Cell. Neurosci.* 4: 12 10.3389/fncel.2010.00012 PMC2876868 20508743

[B35] Valera A. M. Doussau F. Poulain B. Barbour B. Isope P. (2012). Adaptation of granule cell to Purkinje cell synapses to high-frequency transmission. *J. Neurosci.* 32 3267–3280 22378898 10.1523/JNEUROSCI.3175-11.2012 PMC6622027

